# The External Application of Sulphur in Sciatic Neuralgia

**Published:** 1888-08

**Authors:** J. W. Cowden

**Affiliations:** Rock Island, Ill.


### THE EXTERNAL APPLICATION OF SULPHUR IN SCIATIC NEURALGIA.

BY J. W. COWDEN, M. D., ROCK ISLAND, ILL.

(Read at the Thirty-eighth Annual Meeting of the Illinois Sta e Medical Society, May 17, 1888.)

Sulphurous baths, natural and artificial, have been in vogue for a long time in the treatment of rheumatism and neuralgia. The external application of the flowers of sulphur, however, in the treatment of sciatic rheumatism, although its therapy, on second thought from our previous knowledge of the action of sulphur, would seem rational enough, has but recently been tried.

In the *Therapeutic Gazette*, for April, 1888, will be found an article on the "External Application of Sulphur in Sciatic Neuralgia." In confirmation of what is therein stated, I beg leave to report to this society the following cases which recently came under my observation:

CASE.—J. R., aged 45, weighing 180 pounds, an Irishman of sanguine temperament and strong constitution, in robust health up to the time of this attack; saloon-keeper by occupation, very happy in his calling, and cheerfully waited upon his customers. For two months before I was called to see him, he had been an almost constant sufferer from sciatic neuralgia. He finally became unable to walk about, was taken to his bed, and to use his own language at the time I first saw him, "Doctor, I am suffering the tortures of the d——d and am not able to sit, stand nor lie." He had a haggard, worn look, and his condition was, indeed, pitiable in the extreme. For some four weeks the usual remedies for neuralgia were tried with indifferent results, until finally, in addition to the usual treatment it took, morphine sulphas, gr. ss atropiæ sulphatis, gr. 1-60, hypodermically twice or three times in twenty-four hours, to give him even temporary relief from his horrible suffering. At the end of this time I called one morning and found him in agony, writhing in torture and completely discouraged. He begged most pitifully for the hypodermic injection and said he would die. I told him I would give him no more hypodermics and no more medicines, but would bury him in sulphur instead. He then said, "My God, Doctor, I will die before night if I do not get relief." I then told him that if the sulphur did not relieve him he would have to die, as that would be the only treatment for the next twenty-four hours. The limb was accordingly enveloped in the dry sulphur. In less than two hours he was sweating profusely, sleeping soundly and oblivious of all pain and suffering. He roused up in the evening long enough to take some nourishment, then again fell asleep and slept continuously during the following night, the perspiration continuing, and awoke in the morning free from pain, able to turn over in bed and move and extend the limb in all directions without complaint. The look of suffering which had been so marked before the application of the sulphur was gone. He got up, and to his great surprise walked easily about the room without suffering or pain. He was then put into a large wash-tub, thoroughly scrubbed and washed with soap and water, after which, at his own request, he was again put to bed and the sulphur re-applied to the limb and sacral region of the spine. The next morning he was given another bath, the neuralgia had disappeared, and from that time without further medication his recovery was continuous, and, as far as the pain is concerned, up to this writing is complete. For a few days after discontinuing the sulphur he suffered from sleeplessness and nervous prostration, but the further progress of the case towards recovery was left, as Bayard would say, "to the *vis medicatrix naturæ*." The perspiration, the breath and the urine after the application of the sulphur were very soon impregnated with sulphuretted hydrogen, making it very disagreeable for the patient and his attendants on that account. The rapid absorption of the sulphur, as shown by the profuse perspiration, perfumed breath, etc., and the speedy relief which followed its application, would seem to point to a specific action of the remedy.

*Supplement.*—On the 30th day of April, some two weeks after the above report was written, the patient had a relapse, caused by sleeping in a draft between two open windows, so that the sulphur had to be re-applied. The same happy effect, entire relief from his pain, followed as quickly and promptly as it did on the first application of the remedy.

On the 10th of May, in consequence of his anæmic condition caused by his long sickness, I put him on 20-drop doses of the tincture of iron four times a day, since which time he has rapidly gained in health, and is now entirely free from his neuralgia.

"There is a pleasure in the pathless woods of speculative medical philosophy; a rapture on the lonely shores of the imagination," of the disconcerted doctor, known only to certain medical minds so formed as to be ever open to the reception of theoretical ideas and impressions for the relief of suffering humanity. It may yet prove to be the most happy conception, freighted with relief for suffering mortals, which suggested to the imagination of the enthusiastic speculative medical philosopher the idea of applying sulphur externally in sciatic neuralgia, so quickly and speedily does entire relief from horrrible suffering follow the application.

